# Energy transfer in reactive movements as a function of individual stretch load

**DOI:** 10.3389/fphys.2023.1265443

**Published:** 2023-11-30

**Authors:** Janice Waldvogel, Kathrin Freyler, Ramona Ritzmann, Albert Gollhofer

**Affiliations:** Department of Sport and Sport Science, University of Freiburg, Freiburg, Germany

**Keywords:** electromyography, fascicle, muscle-tendon unit, operating length, stretch-shortening cycle

## Abstract

**Background:** By directly recording electromyographic activity profiles and muscle-tendon interaction, this study aimed to elucidate the mechanisms why well-trained track and field athletes (experts) are able to outperform untrained individuals without former systematic experience in reactive jump training (novices). In particular, reactive power output and the elastic recoil properties of the muscle-tendon unit (MTU) were of special interest. For this purpose, stiffness regulation on muscle and joint level, energy management in terms of storing or dissipating elastic energy were compared between experts and novices during various stretch loads.

**Methods:** Experts were compared with novices during reactive drop jumps (DJs) from drop heights ranging between 25 and 61 cm. Delta kinetic energy (Ekin) was calculated as the difference between the Ekin at take-off and ground contact (GC) to determine energy management. By recording electromyography of the lower limb muscles, *in vivo* fascicle dynamics (gastrocnemius medialis) and by combining kinematics and kinetics in a 3D inverse dynamics approach to compute ankle and knee joint kinetics, this study aimed to compare reactive jump performance, the neuromuscular activity and muscle-tendon interaction between experts and novices among the tested stretch loads.

**Results:** Experts demonstrated significantly higher power output during DJs. Among all drop heights experts realized higher delta Ekin compared to novices. Consequently, higher reactive jump performance shown for experts was characterized by shorter GC time (GCT), higher jump heights and higher neuromuscular activity before and during the GC phase compared to novices. Concomitantly, experts were able to realize highest leg stiffness and delta Ekin in the lowest stretch load; however, both groups compensated the highest stretch load by prolonged GCT and greater joint flexion. On muscle level, experts work quasi-isometrically in the highest stretch load, while in novices GM fascicles were forcefully stretched.

**Conclusion:** Group-specific stiffness regulation and elastic recoil properties are primarily influenced by the neuromuscular system. Due to their higher neuromuscular activity prior and during the GC phase, experts demonstrate higher force generating capacity. A functionally stiffer myotendinous system through enhanced neuromuscular input enables the experts loading their elastic recoil system more efficiently, thus realizing higher reactive power output and allowing a higher amount of energy storage and return. This mechanism is regulated in a stretch load dependent manner.

## 1 Introduction

Are highly trained track and field athletes able to tolerate higher stretch loads and consequently develop higher reactive power output in stretch-shortening type actions?

During reactive movements, humans utilize musculoskeletal springs to alternately store and restitute elastic energy (Alexander and Bennet-Clark, 1977; Cavagna, 1977; Komi, 2000). Naturally, in the jump transition, from eccentric to concentric phase, pre-activated muscles and tendons interact, collectively forming an elastic recoil system (Komi, 2000). The efficiency of this elastic recoil system, characterized by high reactive power output and the ability to transfer energy from the pre-activated to the eccentrically stretched muscle-tendon unit (MTU) to the concentric take-off (stretch-shortening cycle, SSC), is determined by the MTU’s stiffness and viscoelastic properties (Taube et al., 2012a). Thereby, spinal and supra-spinal input from the central nervous system (CNS) determines the MTU’s stiffness by modulating 1) the pre-programmed muscle activity prior ground contact (GC), 2) the reflex associated muscle activity and 3) the pre-programmed muscle activity after GC (Taube et al., 2012a).

While early pioneering experiments exploring the mechanisms for force and power potentiation during SSC type actions were particularly biomechanically oriented (e.g., Asmussen and Bonde-Petersen, 1974; Cavagna, 1977), the use of non-invasive methods has significantly expanded the understanding of muscle function during dynamic movements. In particular, the development of surface electromyography but also the later use of electrophysiological stimulation techniques provided crucial evidence for the involvement of stretch reflexes (Leukel et al., 2008a; Leukel et al., 2008b; Zuur et al., 2010), which had previously been controversially discussed (e.g., Komi and Gollhofer, 1997; van Ingen Schenau et al., 1997). In addition, neuromechanical evidence has shown that the contribution of the triceps surae synergists to reactive power output and energy transfer during SSC type movements, predominantly regulated via the ankle joint (e.g., during drop jumps, DJs), should be considered muscle specifically. On the one hand, the mono-articular soleus (SOL) muscle has a high potential for eliciting stretch reflexes and contributing to stiffness regulation due to its spindle equipment (Banks, 2006) and its typical stretch-shortening behavior as evidenced by ultrasound recordings (Sousa et al., 2007). On the other hand, it has been shown that the gastrocnemius medialis (GM) muscle requires a well-tuned muscle activity, in order to work at an almost constant length (Fukashiro et al., 2006). According to the force-velocity relationship such quasi-isometric behavior has been shown for various locomotive movements (e.g., Lichtwark and Wilson, 2005; Fukashiro et al., 2006; Sousa et al., 2007). This muscle-tendon interaction ensures high force generation with optimal tendon loading, thus minimizing the amount of energy transferred to heat (energy dissipation). However, maximizing reactive power output and energy transfer with the aim of storing and releasing elastic energy rather than energy dissipation requires three fundamental conditions: a well-timed pre-activation of the muscles before the eccentric phase, a short and fast eccentric phase and an immediate transition between stretch and shortening (Komi and Gollhofer, 1997).

In this context, both the neuromuscular activity and the muscle-tendon interaction have been shown to be modulated as a function of stretch load (Komi and Gollhofer, 1997; Sousa et al., 2007; Leukel et al., 2008b). Studies have shown that upon an inter-individual stretch load the neuromuscular activity before and during the GC phase is enhanced (Avela et al., 1996; Komi and Gollhofer, 1997; Sousa et al., 2007), which contributes to adequate stiffness regulation (Arampatzis et al., 2001; Hobara et al., 2007). However, when an inter-individually tolerable stretch load is exceeded, the electromyographic activity and stretch reflexes are reduced (Komi and Gollhofer, 1997), thus compromising the muscle-tendon interaction and consequently the energy management in terms of energy storage and release (Komi and Bosco, 1978; Sousa et al., 2007). Such changes have previously been associated with presynaptic inhibition (Leukel et al., 2008a; Leukel et al., 2008b), which consequently impairs MTU’s stiffness and facilitates forceful fascicle stretch (Ishikawa and Komi, 2004; Sousa et al., 2007). If the mechanical stretch load is too high, cross-bridges exceed their limits of the short-range elastic stiffness (SRES) (Rack and Westbury, 1974), thereby compromising their force generating capacity (Ishikawa et al., 2005). Concomitantly, the tendon’s functional role changes from spring-like elastic to a more viscoelastic interaction. Such active buffering is typically observed during pure energy dissipation tasks such as landing or decelerating (Lindstedt et al., 2001; Werkhausen et al., 2017; Waldvogel et al., 2023). The mechanisms of SSC have been studied extensively over the past decades; however, several aspects regarding the technological limitations have to be considered. Technological advances in quantifying muscle function in dynamics have greatly expanded the basic predominantly biomechanical understanding of muscle-tendon interaction. Therefore, studies aimed to investigate stretch load dependent neuromuscular control and muscle-tendon interaction during SSC type movements either in untrained subjects with low to moderate stretch load tolerance (Gollhofer et al., 1992; Ishikawa and Komi, 2004; Lichtwark and Wilson, 2005) or during reactive movement pattern (e.g., counter movement jump, CMJ) that does not necessarily meet the requirements closely related to energy storage processes (e.g. Aeles et al., 2018; Aeles and Vanwanseele, 2019; Werkhausen et al., 2019). From a biomechanical perspective such population kinematically compensates for excessive stretch loads with high ankle and knee joint flexion, which increases the time to decelerate the center of mass (COM) velocity (Minetti et al., 1998), thus compromising the anchoring function of the GM muscle and the energy storage. From the few existing biomechanically orientated studies it is known that highly trained athletes are able to maintain high leg stiffness during moderate (40 cm) and high stretch loads (80 cm) without kinematic compensation via the ankle and knee joint (Viitasalo et al., 1998). Additionally, findings from CMJs, rapid deceleration and from DJs performed within a sledge jump system are particularly noteworthy. Previously, it was shown that both phase-specific electromyographic activity and the morphological MTU’s properties (e.g., tendon stiffness) are modulated by training (Taube et al., 2012b; Alkjaer et al., 2013; Hirayama et al., 2017). Such modulation are of paramount importance because from our understanding, track and field athletes from the sprint and jumping disciplines meet the three fundamental conditions postulated for maximising mechanical power output and energy transfer during reactive movements (Komi and Gollhofer, 1997). Due to the missing systematic studies applying a complex methodological approach, the neuromuscular activity and muscle-tendon interaction during DJs in individuals with very high stretch load tolerance, as it exists in the model of long-term trained athletes, is currently unexplored. Therefore, this study was designed to extend the current mechanisms during the SSC to functionally understand the dependency of stiffness regulation and the associated energy management during distinct stretch loads by directly contrasting experts (high stretch load tolerance) with untrained individuals (further termed as novices, low stretch load tolerance).

It was hypothesized that experts are able to realize higher electromyographic activity in the shank muscles among all tested stretch loads (low to high). Higher electromyographic activity before and during GC was expected to allow the fascicles to work quasi-isometrically. Specifically, when exposed to the highest stretch load, it was hypothesized that experts were able of increasing their MTU’s stiffness through increased neuromuscular input, and consequently, realize highest reactive power output through improved elastic recoil properties. In contrast, novices were expected to exceed their inter-individually tolerable stretch load and thus exhibit less efficient neuromuscular stiffness regulation, resulting in forceful fascicle stretching and impaired energy storage and release. As experts from the track and field disciplines are long-term drilled to generate high propulsive power in a limited amount of time (Seyfarth et al., 1999; Coh and Mackala, 2013) experts were expected to realize shorter GC times (GCT) and higher leg stiffness among all stretch loads. By using an innovative complex methodological approach this study aimed to systematically investigate the global biomechanical regulations among different stretch loads on a muscle tissue basis combined with the underlying neuromuscular compensatory mechanisms between experts and novices.

## 2 Materials and methods

### 2.1 Experimental design

In a cross-sectional study design, the difference in neuromuscular control, the muscle-tendon interaction and the associated energy management was elaborated between national elite athletes and novices. Electromyographic (EMG) activity of the shank muscles of the dominant leg was combined with ultrasound recordings of the GM muscle and three-dimensional (3D) motion analysis. The dominant leg was predefined as the self-reported take-off leg during track and field jumping disciplines.

### 2.2 Participants

Twenty-eight subjects [14 experts (8 males, 6 females), 14 novices (8 males, 6 females)] participated in this study. Experts were elite athletes recruited from the national track and field cadres. Inclusion criteria for experts were: 1) an age above 17 years, 2) competing at national or international level (placed among the 20 best recent results in the track and field jumping and/or sprinting disciplines; Germany) and having at least 3 years’ experience in systematic reactive jump training, 3) portfolio of sprint and jump disciplines, decathlon, or heptathlon. Inclusion criteria for novices were: 1) recreationally engaged in exercise (min 3 h/week), 2) with no former competitive experience in track and field jump categories and, 3) no record of systematic reactive jump training within the last 3 years. They were recruited if they had a reliable jump pattern. Exclusion criteria, for both groups, is described elsewhere (Waldvogel et al., 2021). All participants or their parent or legal guardian gave their written informed consent to the experimental procedure, which was in accordance with the latest revision of the Declaration of Helsinki and approved by the ethics committee of the University of Freiburg (430/17). Their mean (± standard deviation, SD) height, body mass and age were:

• experts: 178 ± 8 cm, 71 ± 10 kg, and 21 ± 4 years
• novices: 176 ± 6 cm, 71 ± 10 kg and 24 ± 4 years

Based on the findings of recent studies (Waldvogel et al., 2021) an *a priori* power analysis (G*Power 3.1.9.7) was conducted (f = 0.25, power = 0.8, α = 0.05).

### 2.3 Procedures

After a standardized warm up (Waldvogel et al., 2021), individuals performed maximal voluntary isometric contractions (MVC) for each recorded muscle for normalization purposes according to procedures described elsewhere (Ritzmann et al., 2016). Thereafter, subjects performed reactive bipedal DJs with both hands placed at the hip (akimbo) from different drop heights adjusted by a hydraulic lifting platform. The familiarization and the testing DJs were performed in a single session. The drop heights were randomly allocated with equidistance ranging from 25 to 37 to 49 to 61 cm. Movement execution was strictly supervised by the authors to avoid potential learning effects and to ensure a reliable jump pattern with stiff knee joints. In total subjects performed 10 jumps per drop height; after 10 consecutive jumps subjects had a 3-min break to avoid fatigue. Subjects were instructed to jump as high as possible and to keep the GCT as short as possible (Kramer et al., 2010).

### 2.4 Data collection and processing

#### 2.4.1 Force

Three-dimensional ground reaction forces (GRF) were recorded separately for the right and left leg with a sampling frequency of 2 kHz (AMTI, OR6-6, Watertown, United States). GRFs [N] from the force plate of the dominant leg were used to detect the event of GC (threshold was set to 20 N), GCT and to calculate peak vertical GRF (F_max_).

#### 2.4.2 Kinematics

A 3D motion capture system (VICON^®^, 10 infrared cameras, Oxford Metrics, Oxford, UK, sampling frequency 200 Hz) recorded full body kinematics from 39 reflective markers (Plug-In-Gait marker set, 14 mm in diameter) (Kramer et al., 2012). To calculate joint kinematics, a conventional gait model was applied (Baker et al., 2018). From the 3D full body model (Plug-in Gait), ankle and knee joint angles were assessed for the sagittal plane. Prior calculating joint angles and joint moments, marker trajectories were filtered with a 4th order Butterworth filter with a cut-off frequency of 15 Hz. Kinematic data was processed by VICON motion analysis software (Nexus 11.0, VICON Motion Systems Ltd., Oxford, UK). External joint moments were calculated in the distal segment coordinate system with a standard inverse dynamic approach integrating kinematic and kinetic data using the Golem model (Wirsching, 2013). Ankle and knee joint power normalized to body mass were calculated as the product of net joint moment and joint angular velocity. The COM deceleration phase was defined as the phase beginning with the initial GC and ending with completing the COM downward movement (COM_min_). The COM trajectory was used to calculate jump height (Wank and Coenning, 2019) and the vertical displacement of the COM during GC until COM_min_, further described as COM displacement. Reactive strength index (RSI) was calculated by dividing jump height by the GCT. Leg stiffness was calculated as the ratio of F_max_ and COM displacement during the time interval from GC until F_max_ (Günther and Blickhan, 2002). Based on the COM’s velocity, kinetic energy (Ekin) was determined for the time points one frame prior to GC (Ekin _GC_) and take off (Ekin _TO_) (51). ∆ Ekin was defined as the difference between Ekin _TO_ and Ekin _GC_, further termed as energy turnover (Waldvogel et al., 2023).

#### 2.4.3 Ultrasonography

GM fascicles were imaged using a B-mode ultrasound device (ArtUs EXT-1H, Telemed, Lithuania) and recorded with a capture frequency of 138 Hz with the corresponding software (Telemed Echo Wave II Vers. 3.6.2, Telemed, Lithuania). The transducer was placed over the medial part of the muscle belly to visualize fascicles and aponeuroses. To avoid probe’s movement above the skin the transducer (96-element, 6cm linear-array probe, 7 MHz, 50 mm imaging depth, 60 mm imaging width) was positioned within an ultralight plastic frame, securely fastened to the skin with adhesive tape at the interface according to Waldvogel et al. (2021). Data was analysed using a semi-automated tracking algorithm (Farris and Lichtwark, 2016). The fascicle length was calculated as the length between the lower and the upper aponeuroses. For the lowest and the highest drop height (25 and 61 cm) the absolute fascicle trajectories for the three trials with the highest RSI were analysed for the time interval ranging from 145 ms before GC until 50 ms after take-off to provide sufficient time buffer for subsequent filtering. Afterwards, trajectories were filtered with a 4th order Butterworth filter with a cut-off frequency of 10 Hz (van Hooren et al., 2020). The trajectories for the three trials were averaged per subject and condition. From the averaged trajectories the absolute fascicle length was determined for the following time points: instant of GC and COM_min_. For the figures the averaged fascicle trajectories per subject and condition were normalized to the time interval from GC ranging until COM_min_. The sarcomere length was theoretically contextualized by the procedures reported by Monti et al. (2021). In detail sarcomere number was calculated by dividing the individual fascicle length while standing in an upright position by the reported sarcomere resting length of 3.09 μm measured in vivo in the same position (Sanchez et al., 2015). The individual fascicle length obtained for the abovementioned time points (GC and COM min) was divided by the reported sarcomere number to obtain an average sarcomere operating length in each time point. Subsequently the sarcomere operating length was superimposed to the force-length relationship described by Walker and Schrodt (1974). According to Hawkins and Hull (1990) MTU length was approximated by using the ankle and knee joint angles and the individual shank length. Fascicle length, sarcomere length and MTU length at the time points of the instant of GC and COM min were used for the statistical analysis (Waldvogel et al., 2023).

#### 2.4.4 EMG

Bipolar Ag/AgCl surface electrodes (Ambu Blue Sensor P, Ballerup, Denmark, diameter 9 mm, center-to-center distance 34 mm) were placed over the soleus (SOL), GM, and the tibialis anterior (TA) of the dominant leg. The longitudinal axes of the electrodes were in line with the presumed direction of the underlying muscle fibers. Inter-electrode resistance was kept below 5 kΩ by means of shaving, light abrasion and degreasing of the skin with a disinfectant. Procedures were executed according to SENIAM (Hermens et al., 2000). The EMG signals were filtered, amplified (band-pass filter 10 Hz to 500 Hz, 200x amplified) and recorded with 2 kHz (A/D-conversion via a National Instruments PCI-6229 DAQ-card, 16bit resolution). The EMG signals during the MVCs were rectified and integrated (iEMG) for each recorded muscle for the time interval ranging from 25 ms before and after the peak value reached during MVC trial. The iEMG signals were further used for data normalization processes (Ritzmann et al., 2016). The EMG signals during reactive DJs were rectified and integrated for the following time intervals: pre-activity phase (100 ms before GC until GC), instant of GC (25 ms before until 25 ms after GC), short-latency response (SLR, 30–60 ms after GC) and the COM deceleration phase (GC until COM_min_). Subsequently, iEMGs were time normalized to 1 s (mVs) to compare the time intervals and then normalized to the respective MVC iEMG (Ritzmann et al., 2016; Helm et al., 2020).

### 2.5 Statistics

Python3 (version 3.6) was used for parameter calculations. To synchronize the methodological devices with different recording frequencies, the whole data set was interpolated to 2 kHz. In two subjects, ultrasound data was excluded from analysis due to insufficient quality. Data is averaged for trials and subjects and presented as grand means ± standard deviation in tables and figures.

The statistical analysis was executed using R studio (R version 4.1.0). To detect interaction effects between group and drop height, a two-level linear mixed model (LMM) (2 × 4; experts/novices and 25/37/49/61) with *a priori* contrasts was applied; p-values were adjusted by Tukey method. “Lme4” R package was used to fit the LMMs. Group and drop height were set as fixed factor, while subjects were factored as random effects. Normality of the residuals was checked visually with QQ-plots. Transformed values are indicated accordingly in the tables if data did not follow normal distribution. The level of significance was set to *p* < .05, and effect size was expressed by partial eta-squared (eta^2^) and calculated by using the R package “effectsize”. Hereby, effect size was interpreted as small (≥0.01 and <0.06), intermediate (≥0.06 and <0.14), and large effects (≥0.14) (Cohen, 1988). Model fit was checked by Akaike-information-criterion (AIC); the model with the lowest AIC is reported in the tables. We started with the saturated model (random slope and random intercept model); in case of not reaching convergence, complexity was systematically reduced during the modelling process.

## 3 Results

### 3.1 Kinetics

Significant group by drop height interaction effects were shown for leg stiffness (*p* < .001, eta^2^ = .02) and F_max_ (*p* < .001, eta^2^ = .2); with experts realizing a higher leg stiffness (+33–56%, *p* < .001) among all drop heights as compared to novices (Table 1). However, experts demonstrated highest leg stiffness in 25 cm and a significantly and progressively reduction from 25 to 61 cm (−15%). In contrast novices maintained their leg stiffness among all drop heights. Experts demonstrated higher F_max_ compared to novices; in both groups F_max_ significantly increased from 25 to 61 cm.

**TABLE 1:** Stretch-shortening cycle performance and spring mass characteristics.

			Cond	Group						Statistics		
	n	Unit	DH	Experts			Novices			G	DH	G*DH
Jump height^log^	28	m	25	0.41^*^	±	0.06	0.31	±	0.03	F (1, 26) = 33.9, *p* < .001, eta^2^ = .57	F (3, 1071) = 17.9, *p* < .001, eta^2^ = .05	F (3, 1071) = 1.1, *p* =.178, eta^2^ < .00
			37	0.42^*^	±	0.06	0.32	±	0.03			
			49	0.43^*^	±	0.06	0.33	±	0.03			
			61	0.43^*†^	±	0.07	0.33^†^	±	0.03			
GCT^log^	28	ms	25	168^*^	±	19	200	±	28	F (1, 26) = 13.0, *p* =.001, eta^2^ = .33	F (3, 1071) = 94.3, *p* < .001, eta^2^ = .21	F (3, 1071) = 1.6, *p* =.332, eta^2^ < .00
			37	171^*^	±	18	200	±	25			
			49	178^*^	±	18	207	±	27			
			61	186^*†^	±	18	221^†^	±	33			
COM displacement	28	m	25	0.10^*^	±	0.02	0.13	±	0.03	F (1, 26) = 13.5, *p* =.001, eta^2^ = .34	F (3, 1071) = 562.3, *p* < .001, eta^2^ = .61	F (3, 1071) = 0.6, *p* =.624, eta^2^ < .00
			37	0.11^*^	±	0.02	0.14	±	0.02			
			49	0.13^*^	±	0.02	0.16	±	0.02			
			61	0.14^*†^	±	0.03	0.17^*†^	±	0.03			
F_max_^log^	28	N	25	2363	±	619	1965	±	333	F (1, 26) = 2.9, *p* =.098, eta^2^ = .10	F (3, 1071) = 132.0, *p* < .001, eta^2^ = .27	F (3, 1071) = 5.6, *p* < .001, eta^2^ = .20
			37	2502	±	578	2170	±	394			
			49	2621	±	576	2337	±	370			
			61	2946^†^	±	577	2790^†^	±	617			
Leg stiffness^log^	28	N/mm	25	26217^*^	±	10472	16829	±	5603	F (1, 26) = 9.0, *p* =.006, eta^2^ = .26	F (3, 1071) = 13.2, *p* < .001, eta^2^ = .04	F (3, 1071) = 7.5, *p* < .001, eta^2^ = .02
			37	23832	±	9532	16246	±	4859			
			49	21307	±	7583	15739	±	4371			
			61	21820^†^	±	7113	16912	±	5542			

Data are presented as grand means ± standard deviation. Jump height, ground contact time (GCT, time interval from the instant of ground contact (GC) until take-off), vertical center of mass (COM) displacement from GC until reaching the minimum vertical COM position, peak ground reaction forces during the GCT (F_max_) and leg stiffness (ratio between F_max_ and COM displacement) are highlighted for experts and novices for drop heights ranging from 25 to 61 cm. Non-normal distributed raw values were transformed by the logarithm function ^log^ for statistical modelling; however, non-transformed raw values are presented for the grand means ± standard deviation. For each variable, a mixed model was applied for group (G), drop height (DH) and the interaction effect of both fixed factors (G * DH). The level of significance was set to *p* < .05. Significant *a priori* contrasts between experts and novices are indicated by asterisks * (*p* < .05); significant differences within the groups between 25 and 61 cm are highlighted by † (*p* < .05).

**FIGURE 1:**
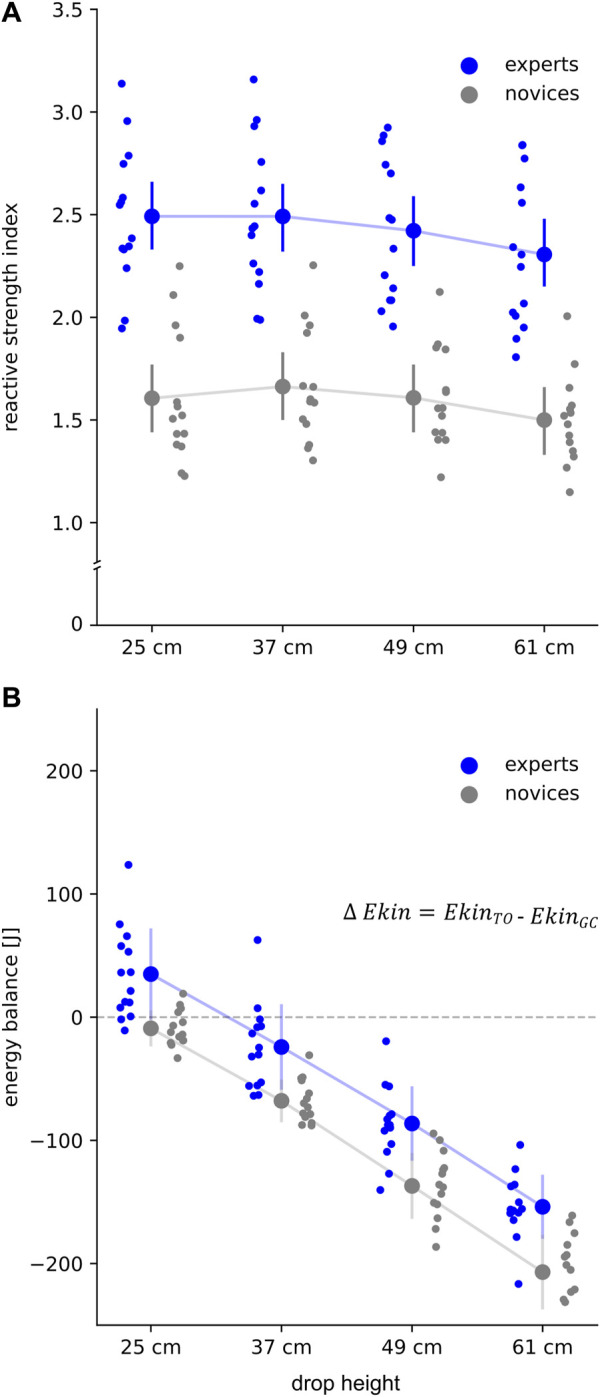
**(A)** Reactive strength index (RSI = jump height divided by ground contact (GC) time) and **(B)** energy balance (delta kinetic energy, ∆ Ekin) for all subjects for each drop height. Grand mean and standard deviation are shown for experts (blue) and novices (grey). Positive energy balance represents higher Ekin at take-off ( ${Ekin}_{TO}$ ) in relation to Ekin at the instant of GC ( ${Ekin}_{GC}$ ), while negative values indicate lower Ekin at TO. The subject’s individual means are shown per drop height in the corresponding color.

Group effect revealed significant higher reactive strength index (+47–56%, *p* < .001, eta^2^ = .67, Figure 1), jump heights (+27–32%, *p* < .001, eta^2^ = .54), combined with significantly shorter GCT (−14%–16%, *p* = .001, eta^2^ = .33) in experts compared to novices. In both groups jump height (EXP +5%, NOV +6%), GCT (EXP +11%, NOV +11%), and F_max_ (EXP +26%, NOV +40%) significantly increased from 25 to 61 cm. Reactive strength index was significantly lower in 61 cm compared to 25 cm (EXP -8%, NOV -6%).

### 3.2 Kinematics and inverse dynamics

Significant group by drop height interaction effects were shown for ankle joint dorsiflexion, positive peak ankle and knee joint power (Table 2). Independently from drop height experts realized significantly lower COM displacement (18%–23%, *p* < .001, eta^2^ = .34), lower ankle joint dorsiflexion and knee joint flexion, significantly higher positive peak ankle and knee joint moments and positive peak ankle and knee joint power as compared to novices. A significant effect for drop height was shown for all dependent variables disregarding groups. Both groups compensated enhanced stretch loads with significantly increased COM displacement (EXP +40%, NOV +31%), ankle joint dorsiflexion and knee joint flexion, peak ankle and knee joint moments and increased negative peak ankle and knee joint power. Positive peak ankle joint power was significantly reduced while positive knee joint power was maintained in both groups.

**TABLE 2:** Ankle and knee joint kinematics and inverse dynamics.

			Condition	Group						Statistics		
	n	Unit	DH	Experts			Novices			G	DH	G*DH
ankle joint dorsiflexion	28	°	25	32^*^	±	5	41	±	7	F (1, 26) = 15.8, *p* < .001, eta^2^ = .38	F (3, 1071) = 470.4, *p* < .001, eta^2^ = .57	F (3, 1071) = 3.4, *p* =.017, eta^2^ <.00
			37	36^*^	±	5	44	±	6			
			49	39^*^	±	5	47	±	6			
			61	41^*†^	±	5	49^†^	±	6			
peak ankle joint moment	28	Nm kg^-1^	25	4.1	±	0.7	3.5	±	0.7	F (1, 26) = 3.3, *p* =.079, eta^2^ = .11	F (3, 1071) = 44.0, *p* < .001, eta^2^ = .11	F (3, 1071) = 1.4, *p* =.25, eta^2^ <.00
			37	4.3	±	0.8	3.8	±	0.7			
			49	4.4	±	0.7	3.9	±	0.7			
			61	4.5^†^	±	0.8	4.0^†^	±	0.8			
negative peak ankle joint power	28	W kg^-1^	25	−25	±	5	−21	±	6	F (1, 26) = 1.7, *p* =.209, eta^2^ = .06	F (3, 1071) = 550.7, *p* < .001, eta^2^ = .61	F (3, 1071) =0.5, *p* =.709, eta^2^ =.00
			37	−31	±	6	−28	±	6			
			49	−35	±	6	−32	±	8			
			61	−39^†^	±	7	−36^†^	±	8			
positive peak ankle joint power	28	W kg^-1^	25	29^*^	±	4	23	±	5	F (1, 26) = 21.5, *p* < .001, eta^2^ =.45	F (3, 1071) = 57.9, *p* < .001, eta^2^ = .14	F (3, 1071) = 4.5, *p* =.004, eta^2^ =.01
			37	30^*^	±	6	22	±	5			
			49	29^*^	±	5	21	±	4			
			61	26^*†^	±	6	17^†^	±	3			
knee joint flexion	28	°	25	19	±	8	28	±	8	F (1, 26) = 9.1, *p* =.006, eta^2^ = .26	F (3, 1071) = 148.4, *p* < .001, eta^2^ = .29	F (3, 1071) = 0.4, *p* =.778, eta^2^ < .00
			37	23	±	8	30	±	8			
			49	26	±	7	34	±	7			
			61	30^†^	±	7	38^†^	±	7			
peak knee joint moment	28	Nm kg^-1^	25	3.6^*^	±	7	2.5	±	5	F (1, 26) = 26.1, *p* < .001, eta^2^ = .50	F (3, 1071) = 216.3, *p* < .001, eta^2^ = .38	F (3, 1071) = 1.4, *p* =.272, eta^2^ <.00
			37	3.9^*^	±	6	2.8	±	6			
			49	4.2^*^	±	5	3.1	±	6			
			61	4.4^*†^	±	5	3.4^†^	±	7			
negative peak knee joint power	28	W kg^-1^	25	−13	±	4	−11	±	3	F (1, 26) = 1.3, *p* =.271, eta^2^ = .05	F (3, 1071) = 1076.3, *p* < .001, eta^2^ = .75	F (3, 1071) =1.1, *p* =.349, eta^2^ <.00
			37	−17	±	4	−15	±	4			
			49	−22	±	4	−21	±	5			
			61	−29^†^	±	5	−27^†^	±	5			
positive peak knee joint power	28	W kg^-1^	25	22^*^	±	5	15	±	3	F (1, 26) = 34.3, *p* < .001, eta^2^ = .57	F (3, 1071) = 7.8, *p* < .001, eta^2^ = .02	F (3, 1071) = 4.5, *p* =.004, eta^2^ <.00
			37	23^*^	±	4	16	±	3			
			49	23^*^	±	3	16	±	3			
			61	23^*^	±	4	16	±	3			

Data are presented as grand means ± standard deviation. Ankle dorsiflexion and knee joint flexion is determined for the time interval from the instant of GC (GC) until reaching the minimum vertical center of mass position (COM _min_). Peak ankle and knee joint moment and power values are determined during the ground contact time ranging from GC until take-off. Ankle and knee joint moments and powers are normalized for body mass. For each variable, a mixed model was applied for group (G), drop height (DH) and the interaction effect of both fixed factors (G * DH). The level of significance was set to *p* < .05. Significant *a priori* contrasts between experts and novices are indicated by asterisks ^*^ (*p* < .05); significant differences within the groups between 25 and 61 cm are highlighted by ^†^ (*p* < .05).

### 3.3 Fascicle and muscle-tendon unit dynamics

**FIGURE 2:**
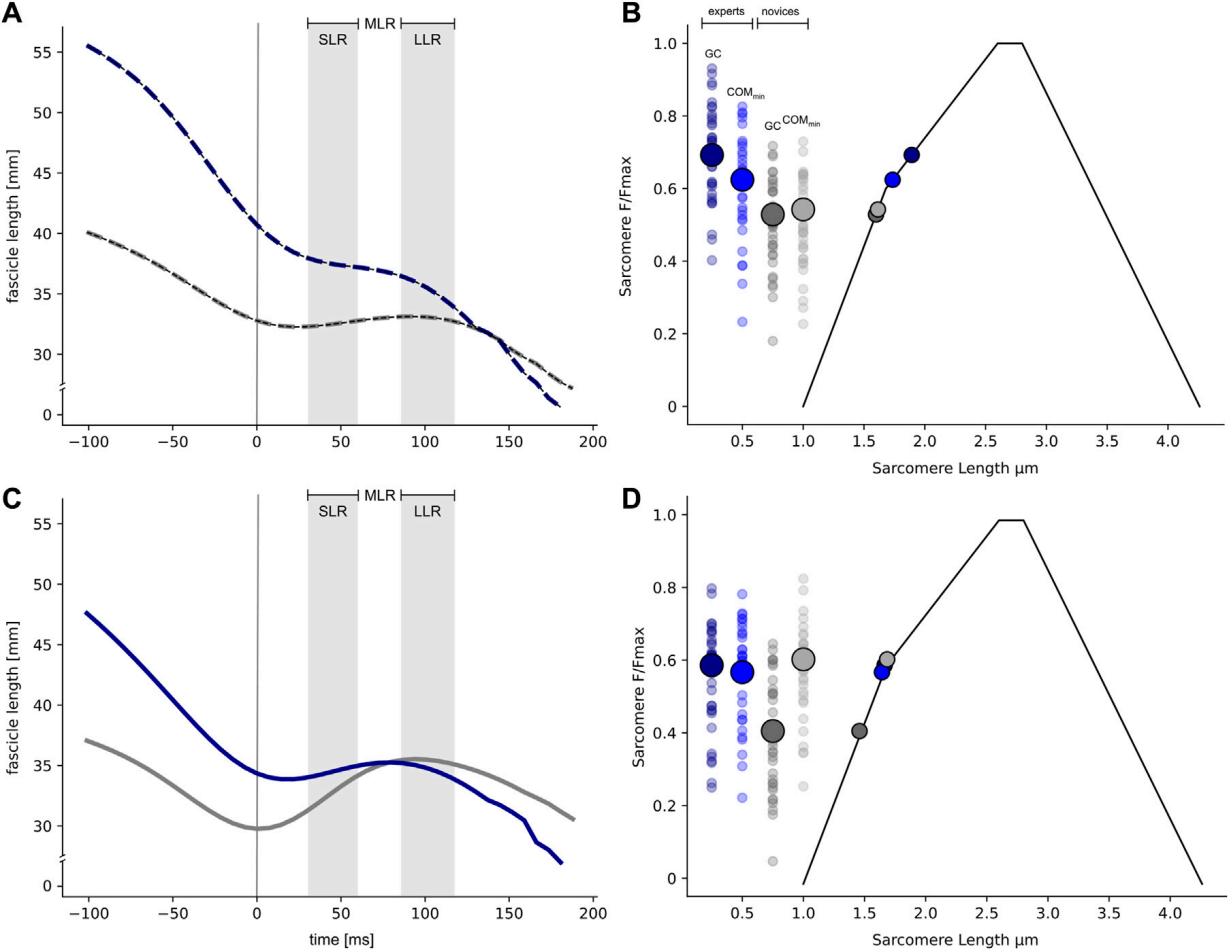
Average fascicle dynamics are shown for experts (dark blue) and novices (grey) separately for the low (A: 25 cm) and high stretch load (C: 61 cm) for the time interval ranging from 100 ms before ground contact (GC) until take-off. The vertical line indicates the instant of GC **(A,C)**, while the time intervals for short- (SLR, 30–60 ms after GC), the medium- (MLR, ranging from 60 to 85 ms after GC) and the long-latency response (LLR, 85–120 ms after GC) are highlighted, respectively. Standard deviations are omitted for clarity. Average operating sarcomere lengths (x-axis) are shown for experts (blue) and novices (grey). Sarcomere lengths are plotted along the force-length relationship (adapted with permission from Walker and Schrodt (1974)) for the instant of GC and the minimum vertical position of the center of mass (COM_min_), separately for the low **(B)** and high stretch load **(D)**.

Regarding the fascicle length significant interaction effects were separately detected for 25 cm (F (1, 120) = 16.2, *p* < .001, eta^2^ = .11) and 61 cm (F (1, 120) = 29.2, *p* < .001, eta^2^ = .2). In 25 cm drops, experts significantly shortened (−10%) their fascicles during the COM deceleration phase, whereas novices demonstrated quasi-isometric fascicle behavior (Figures 2A, C). When jumping from a 61 cm drop height, experts maintained their fascicle length during the deceleration phase, whereas novices clearly demonstrated significant fascicle lengthening (+17%).

In Figures 2B, D the operating sarcomere lengths are shown for the two time points of interest (instant of GC and COM min) for the 25 cm and 61 cm drop heights. Significant interaction effects are observed for 25 cm (F (1, 126) = 13.3, *p* < .001, eta^2^ = .10) and 61 cm (F (1, 120) = 30.4, *p* < .001, eta^2^ = .2). At the instant of GC, experts work closer to their theoretical optimal sarcomere length at a drop height of 25 cm than novices, as evidenced by a significantly longer sarcomere length at the instant of GC. Furthermore, the sarcomere length of experts is shifted towards the lower part of the ascending limb of the force-length relation from the instant of GC (1.91 ± 0.24 μm) until COM_min_ (1.76 ± 0.22 μm) is reached (Figure 2B). In contrast, in novices, the significantly shorter sarcomere length at the instant of GC (1.59 ± 0.16 μm) remains its operating length until COM_min_ (1.59 ± 0.16 μm) is reached. In contrast, the opposite behavior is observed at a drop height of 61 cm. Similar to the 25 cm drop height, at 61 cm the sarcomere length at the instant of GC is significantly shorter in novices (1.45 ± 0.18 μm) compared to experts (1.66 ± 0.2 μm). According to the isometric fascicle behavior observed in experts during the COM deceleration phase, the theoretically estimated sarcomere length remained an almost constant length (COM_min_: 1.68 ± 0.19 μm), which is closer to the plateau of the force-length relationship, indicating a higher theoretical force generating capacity. In contrast, the sarcomere length of novices is shifted towards the upper part of the force-length relationship due to forceful fascicle stretching (1.68 ± 0.19 μm).

Significant interaction effects were detected for the MTU length for 25 cm [F (1, 126) = 17.3, *p* < .001, eta^2^ = .12] and 61 cm [F (1, 120) = 6.9, *p* = .009, eta^2^ = .05]. Both groups demonstrated significant MTU lengthening from the instant of GC until COM_min_, however, the behavior was not significantly different between the groups [25 cm: EXP: 0.43 ± 0.3 m (GC) to 0.46 ± 0.3 m (COM_min_), NOV: 0.41 ± 0.2 m (GC) to 0.44 ± 0.2 m (COM_min_); 61 cm: EXP: 0.43 ± 0.2 m (GC) to 0.46 ± 0.3 m (COM_min_), NOV: 0.41 ± 0.2 m (GC) to 0.44 ± 0.2 m (COM_min_)].

### 3.4 Electromyographic activity during the pre-activity phase

A significant interaction effect was only shown for SOL demonstrating significant higher SOL pre-activity in experts compared to novices among all drop heights (Table 3). In both groups pre-activity of the shank muscles significantly increased as a function of increased drop height. In particular, novices demonstrate significant higher SOL electromyographic activity increase (+36%) than experts (+9%).

**TABLE 3:** Pre-activity of the shank muscles.

	Condition	Group						Statistics		
	DH	Experts			Novices			G	DH	G*DH
TA^sqrt^	25	22	±	10	21	±	10	F (1, 26) = 0.8, *p* =.678, eta^2^ < .00	F (3, 1071) = 48.0, *p* < .001, eta^2^ = .12	F (3, 1071) = 0.9, *p* =.425, eta^2^ <.00
	37	24	±	10	21	±	7			
	49	26	±	11	23	±	8			
	61	28^†^	±	12	26^†^	±	10			
SOL^sqrt^	25	49^*^	±	22	21	±	11	F (1, 26) = 13.8, *p* =.001, eta^2^ = .35	F (3, 1071) =58.8, *p* < .001, eta^2^ = .14	F (3, 1071) =11.6, *p* < .001, eta^2^ =.03
	37	51^*^	±	26	23	±	11			
	49	50^*^	±	21	28	±	15			
	61	54^†^	±	20	33^†^	±	18			
GM^sqrt^	25	81	±	34	73	±	34	F (1, 26) = 0.5, *p* =.497, eta^2^ = .02	F (3, 1071) =35.9, *p* < .001, eta^2^ = .09	F (3, 1071) =1.4, *p* =.105, eta^2^ <.00
	37	84	±	33	79	±	34			
	49	89	±	34	83	±	37			
	61	93^†^	±	34	88^†^	±	35			

Data are presented as grand means ± standard deviation. Integrated electromyographic (iEMG) activity is shown for the pre-activity phase ranging from 100 ms before until the instant of ground contact (GC). IEMGs are shown for the tibialis anterior (TA), soleus (SOL) and gastrocnemius medialis (GM) muscles; values are normalized to the maximum voluntary contraction (% MVC) for each muscle, respectively. Non-normal distributed raw iEMG values were transformed by square root ^sqrt^ for statistical modelling; however, non-transformed raw values are presented for the grand means ± standard deviation. For each variable, a mixed model was applied for group (G), drop height (DH) and the interaction effect of both fixed factors (G * DH). The level of significance was set to *p* < .05. Significant *a priori* contrasts between experts and novices are indicated by asterisks ^*^ (*p* < .05); significant differences within the groups between 25 and 61 cm are highlighted by ^†^ (*p* < .05).

### 3.5 Electromyographic activity during the ground contact phase

**FIGURE 3:**
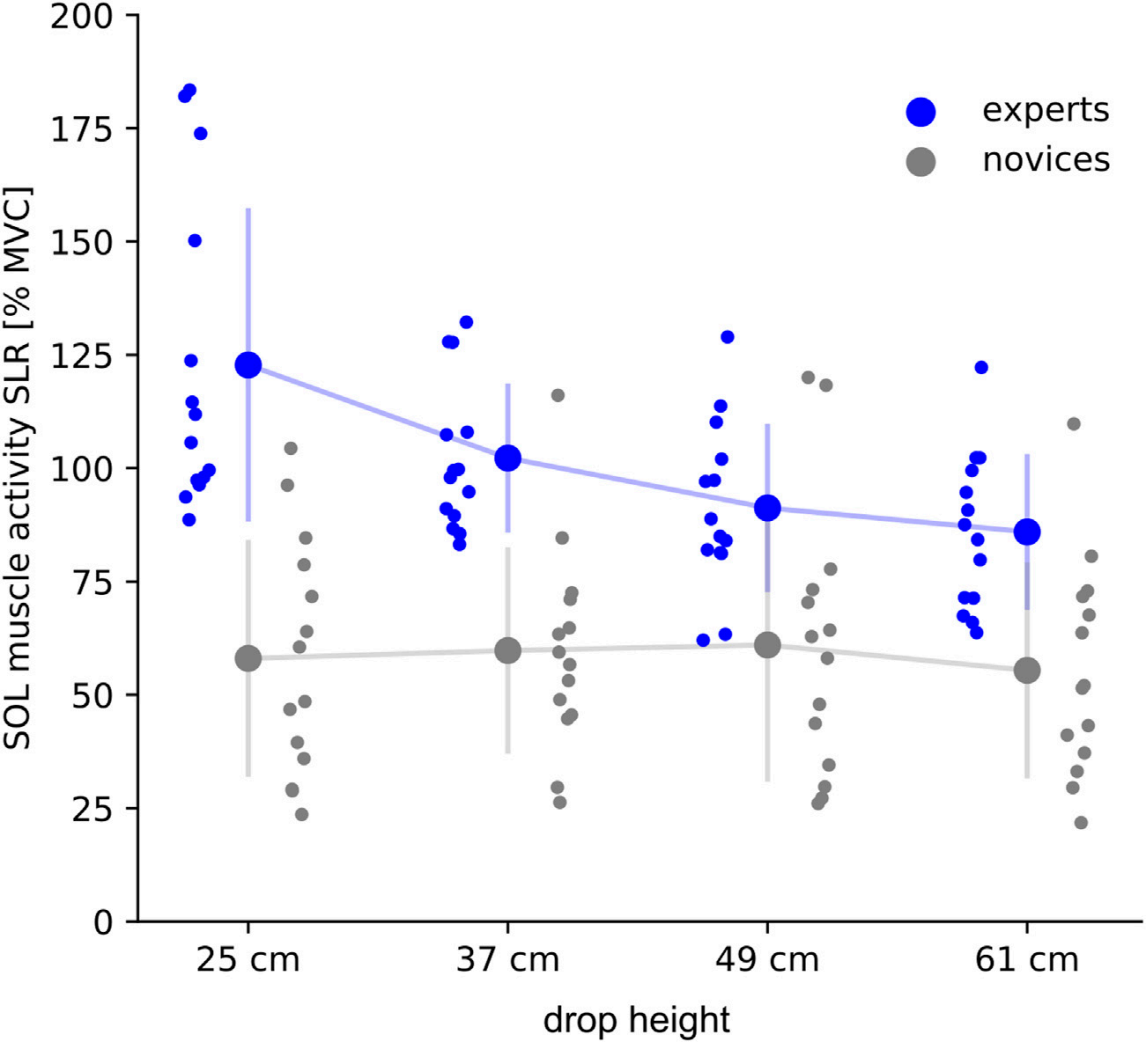
Averaged integrated electromyographic activity (iEMG) is shown for the soleus muscle (SOL) for the short-latency response (SLR, lasting from 30 ms to 60 ms after ground contact) during drop heights ranging from 25 to 61 cm. IEMGs are normalized to the maximum voluntary contraction (% MVC). Grand mean and standard deviation are shown for experts (blue) and novices (grey). The subject’s individual means are shown per drop height in the corresponding color.

Significant interaction effect was only shown for SOL (Figure 3). Experts realized a significantly higher SOL activity during the SLR (25 cm: +46%, 37 cm: +32%) and COM deceleration phase (25 cm: +56%, 37 cm: +49%) compared to novices. GM activity was higher in experts compared to novices but did not reach significance (SLR: +1–5%; COM deceleration phase: +1–2%); for TA lower muscle activity was observed for experts compared to novices during the SLR (−3–17%), while slightly higher values were reported for the COM deceleration phase (+4–14%), no significant differences were detected.

During the GC phase, expert’s SOL muscle activity was significantly reduced from 25 to 61 cm, whereas novices realized similar muscle activity among the drop heights. For all shank muscles significant main effects for drop height were shown independently for group; a high effect size was detected for GM [F (1, 1071) = 59, *p* < .001, eta^2^ = .14]. For both groups, muscle activity of the plantar flexor muscles (SOL and GM) was reduced with increasing drop height; for TA, the neuromuscular muscle activity was systematically increased as a function of drop height.

## 4 Discussion

The main finding of this study is that experts demonstrated a higher reactive power output and energy turnover as compared to novices among all drop heights. Among all drop heights experts realized shorter GCTs, higher jump heights and higher electromyographic activity before and during GC compared to novices. Concomitantly, leg stiffness and energy turnover was highest during low stretch load; however, both groups compensated high stretch loads by prolonged GCTs and higher joint flexion. Moreover, on the muscle level, experts work quasi-isometrically, while novices’ GM fascicles were forcefully stretched during the highest stretch load. Based on the theory-driven mechanistic model presented in the introduction, the stiffness regulation and energy management in the drop height paradigm between experts and novices is integratively considered from a neuromechanical perspective. Particular emphasis is placed on the neuromuscular stiffness regulation, the muscle-tendon interaction and their functional consequences. Consequently, this model is functionally related to the assessment of the energy management during explosive reactive movements (e.g. DJs). The mechanistic model was applied in the context of variable stretch loads and expertise; due to the integrative complexity, results are discussed in detail separately for the low and high stretch load.

### 4.1 Group specific stiffness regulation and energy management during low stretch loads

Based on the neuromechanical evidence, the results suggest that experts are able to mechanically load their elastic recoil system more efficiently during the deceleration phase compared to novices, due to their higher activation profile prior and during GC and due to their active ankle and knee joint control (Lazaridis et al., 2010). Previously it was shown that high pre-programmed muscle activity affects ankle joint stiffness (Farley and Morgenroth, 1999), leg stiffness (Arampatzis et al., 2001; Hobara et al., 2007) and the reflex contribution after GC (Hobara et al., 2007; Taube et al., 2008; Zuur et al., 2010). Functionally, short-term training studies with low stretch loads have shown to effectively enhance the reflex associated neuromuscular activity during the GC phase (Voigt et al., 1998; Taube et al., 2012b; Alkjaer et al., 2013). Based on such empirical findings, the results of the present study indicate that experts were possibly able to integrate their stretch reflexes to a greater extend, thus enhancing their muscle force (Gollhofer et al., 1984) compared to novices. The present results elucidate that the superior reactive power output was achieved by different working ranges in relation to optimal sarcomere lengths. Higher force generating capacity, observed in experts, could rely on more attached cross-bridges, which allow higher SRES and possibly enhanced titin stiffness compared to novices. Recent studies have demonstrated unequivocally that titin stiffness increased upon muscle activation (Lindstedt and Nishikawa, 2017) and thus working as an activation-dependent muscle spring (Nishikawa, 2020) generating mechanical work (Eckels et al., 2018). Therefore, it could be speculated that direct influences of the contractile apparatus due to possible effects of the residual force enhancement (RFE) could play another important role in the SSC. Fukutani et al. (2015) have shown that RFE was higher in situations, when the delay between stretch and shortening was minimized. Therefore, distinct differences observed in experts compared to novices might point towards a common explanation: due to higher electromyographic activity before and during GC and due to the well adapted viscoelastic properties, the experts are able to activate their contractile machinery more efficiently allowing intense muscle stiffness and tolerating high stress to the muscle-tendon complex. Consequently, it may be speculated that experts were able to achieve higher MTU shortening velocities due to their more efficient stiffness regulation on joint and muscle level, further explaining the observed superior energy storage and release and consequently enhanced SSC performance compared to novices. However, it should be emphasized that this unique stiffness regulation is particularly pronounced at low stretch loads; in higher stretch loads such regulation would lead to exceeding loading of the elastic recoil system.

### 4.2 Group specific stiffness regulation and energy management during high stretch loads

As a functional consequence the existing neuromechanical findings implies, that, based on an individual level or disposition, an effective loading of the MTU is limited to a certain amount of stretch load tolerance. As a consequence, reduced neuromuscular pre- and reflex activities, facilitates enhanced joint flexion amplitudes, prolonged GCTs and reduced reactive power output. With reference to such theory-based model, the hypotheses were mostly confirmed for the highest stretch load by the experimental data. Due to their significantly higher pre-programmed muscle activity before and at the instant of GC, it could be conjectured that even during high stretch loads experts are able to additionally potentiate their neuromuscular activity during the deceleration phase due to facilitated stretch reflexes in the SOL muscle elicited by enhanced stretch velocity (Gollhofer and Rapp, 1993). Early experiments have shown unique neuromuscular activity in highly trained experts, while performing reactive DJs (Schmidtbleicher and Gollhofer, 1982). By directly comparing the neuromuscular activity between elite experts with untrained controls a higher neuronal drive during the GC phase was shown for experts dropping from 0.5 m height (Schmidtbleicher and Gollhofer, 1982). Excessive stretch loads when dropping from 1.1 m reduced the neuronal drive to the plantar flexors during the deceleration phase only in untrained subjects, while experts were still able to realize high muscle activity without altered neuromuscular activity (Schmidtbleicher and Gollhofer, 1982). Similar neuromuscular findings were reported for highly trained triple jumpers compared to active controls (Viitasalo et al., 1998). In contrast, the present results indicate significantly reduced muscle activity after GC in experts at high compared to low stretch loads. Similar neuromuscular compensations have recently been reported for hyper-gravity-induced overload (Waldvogel et al., 2021; Waldvogel et al., 2023) and excessive drop heights (Komi and Gollhofer, 1997; Leukel et al., 2008b). This might have several implications: through their active knee joint control, possibly reinforced by the continuous verbal instruction from the operators (“keep your knees as stiff as possible”), the measured experts were still able to maintain a stiff ankle and knee joint throughout the deceleration phase. However, a stiffer joint control and simultaneously enhanced neuromuscular input would mechanically increase the stress on the MTU, which is potentially associated with a higher risk to exceed the muscle and tendon safety factors (Biewener, 2005). During conditions exceeding the individual stretch load tolerances it is argued that inhibitory mechanisms play a progressively more important role (Komi and Gollhofer, 1997; Leukel et al., 2008b). Overall experts demonstrated higher stretch load tolerance than novices.

In novices, reduced muscle activity before and during stretch reflex associated latencies (Leukel et al., 2008b), reduced joint stiffness (Helm et al., 2019), and forceful fascicle stretch (Sousa et al., 2007) are typically associated with energy dissipation (Waldvogel et al., 2023) induced by the CNS throughout inhibitory influences. Regarding the muscle level, the fascicle data have confirmed that mechanical stretch load is too high in novices. Consequently, due to insufficient muscle contraction, cross-bridges possibly are detached beyond their SRES (Ishikawa et al., 2005) resulting in lower force generating capacity evidenced by the theoretical sarcomere contextualization. Similarly, during hyper-gravity-induced overload it was shown that the estimated sarcomere operating range shifts towards the ascending limb (Monti et al., 2021). However, it has been shown that forces similar to those at 1 g can be generated after the deceleration phase (Monti et al., 2021), suggesting that mechanically detached cross-bridges may reattach more rapidly during the take-off phase (Ishikawa et al., 2005). Therefore, the data implies that lower pre-activity combined with reduced reflex associated muscle activity (Helm et al., 2020) potentially impairs additional muscle generated contractile force (SRES and RFE effect) due to titin folding (Eckels et al., 2018).

## 5 Limitations

We acknowledge that this study has some limitations. First, the sarcomere length was contextualized based on fascicle data recorded by ultrasound (Monti et al., 2021). As existing technology to directly measure sarcomere length *in vivo* is invasive (Llewellyn et al., 2008; Sanchez et al., 2015; Lichtwark et al., 2018) and is not yet applicable to dynamic movements, the theoretically derived contextualisation was the only way to get an detailed insight into the force-length relationship on the sarcomere level, however the results only represents average sarcomere behaviour, therefore allowing only limited conclusion. Additionally, it has to be acknowledged that the estimated theoretical sarcomere maximum force generating capability using the force-length relationship proposed by Walker and Schrodt (1974) could have limited validity. Second, arguments related to RFE and titin must be viewed with caution, as they are predominantly based on isolated single fiber studies and therefore are not necessarily valid in the intact muscle model. Nevertheless, in future it would be highly interesting to prove the proposed mechanisms theoretically derived from isolated single-fibre experiments with newly developed technologies specifically designed for dynamic movements.

## 6 Conclusion and perspective

From a mechanistic perspective, the present results emphasized that a pre-activated and highly activated muscle potentially influenced by stretch reflexes, numerous active cross bridges and well adapted tendons found the basis of a highly efficient elastic recoil system. Contrasting the effect of stretch load during reactive DJs between experts and novices differing in their individual stretch load tolerance, the present study aimed to elucidate group-specific differences, particularly pronounced at the neuromuscular and muscle mechanical level. The long-term trained athletes rely on a higher reactive power output and a higher energy turnover largely determined by the immediate transition from the eccentric to concentric contraction. A collectively higher pre-programmed muscle activity triggers higher reflex activity during the GC phase in the model muscle SOL. At the same time, a quasi-isometric GM behavior, which is mainly characterized by higher neuromuscular input and a higher force generating capacity, ensures a more efficient elastic recoil system. Such a higher level and perfectly tuned contractile machinery could also involve titin as a potential player, but its specific role in SSC is still unclear. In addition to theoretically derived arguments, future advances in technology should allow direct investigation of its role during SSC in the intact muscle model.

## Data Availability

The raw data supporting the conclusions of this article will be made available by the authors, without undue reservation.
